# Supplementary figures and images for: Two transcriptionally distinct pathways drive female development in a reptile with both genetic and temperature dependent sex determination
Source: PLoS Genet. 2021 Apr 15;17(4):e1009465. doi: 10.1371/journal.pgen.1009465 (PMC8049264; doi:10.1371/journal.pgen.1009465)

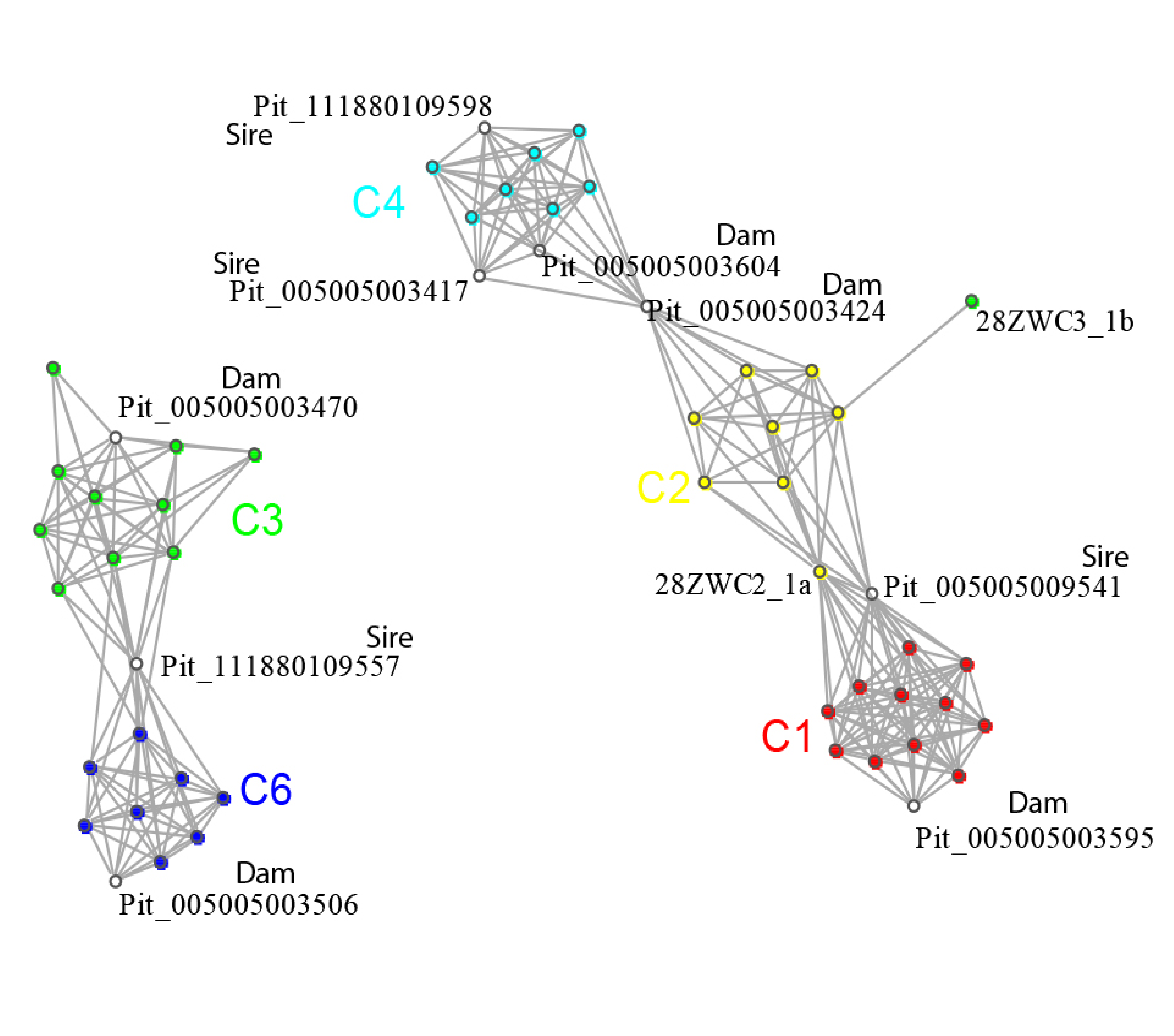

Supplement: S2 Fig — SNP data was generated by Dart sequencing, a reduced genome representation sequencing method at Diversity Arrays Technology, University of Canberra. (TIF) [file pgen.1009465.s013.tif]

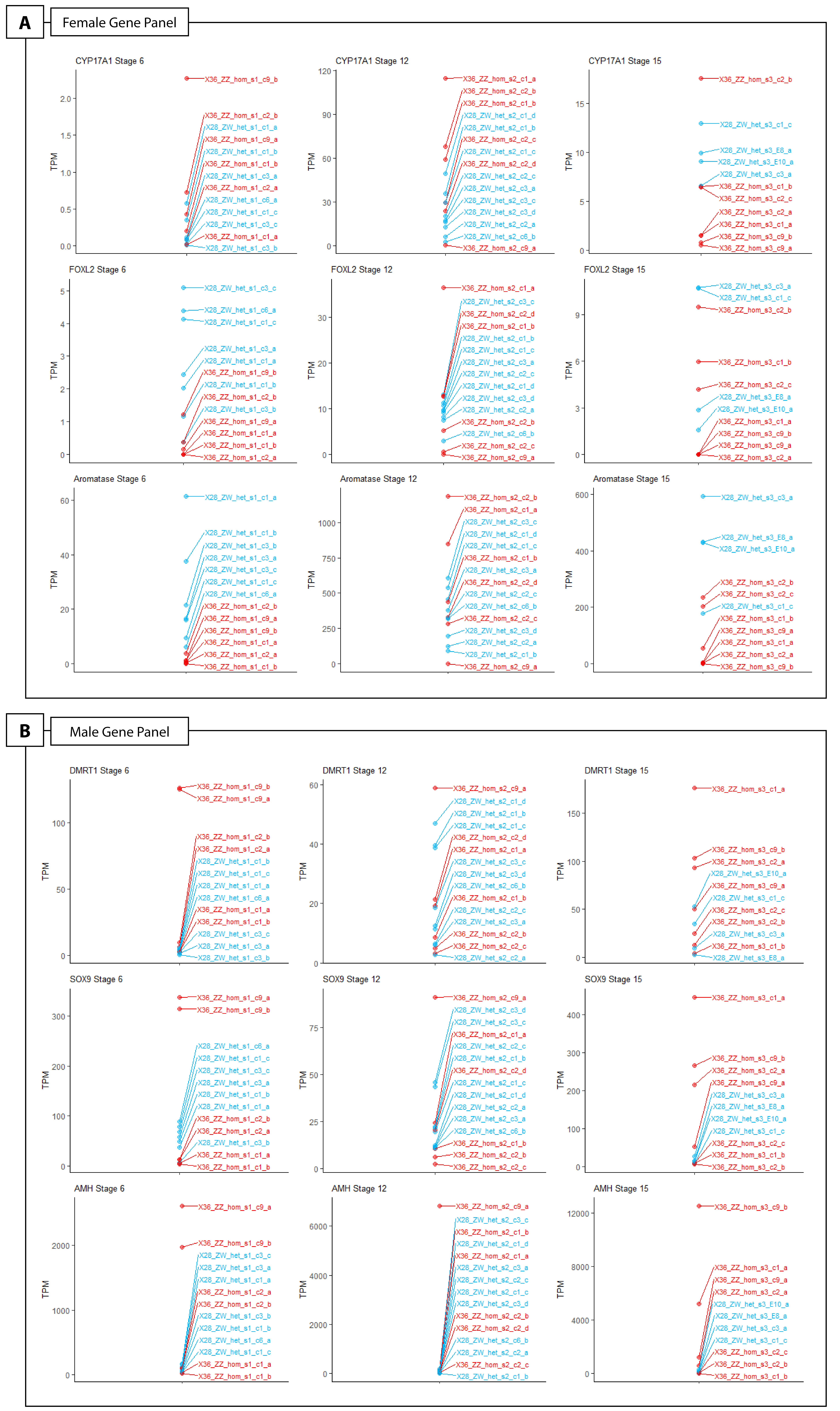

Supplement: S3 Fig — Expression (TPM, transcripts per million) of female-specific genes (CYP17A1, FOXL2, CYP19A1; panel (A) and male-specific genes (DMRT1, SOX9, AMH; panel (B) across three developmental stages (6, 12, 15) [19,20] for all samples to aid in the identification of samples with aberrant expression patterns. Samples from later developmental stages that exhibit low expression of female-specific genes are likely to have not undergone sex reversal. Sample ID labels correspond to incubation temperature (36°C or 28°C in red or blue respectively), maternal genotype/maternal homozygosity/maternal heterozygosity (ZZf or ZWf), sample stage (s1 = stage 6, s2 = stage 12, s3 = stage 15), clutch number (c1, c2, etc.), and replicate ID (e.g., “a” denotes the sample was the first replicate for that sampling point). (TIF) [file pgen.1009465.s014.tif]

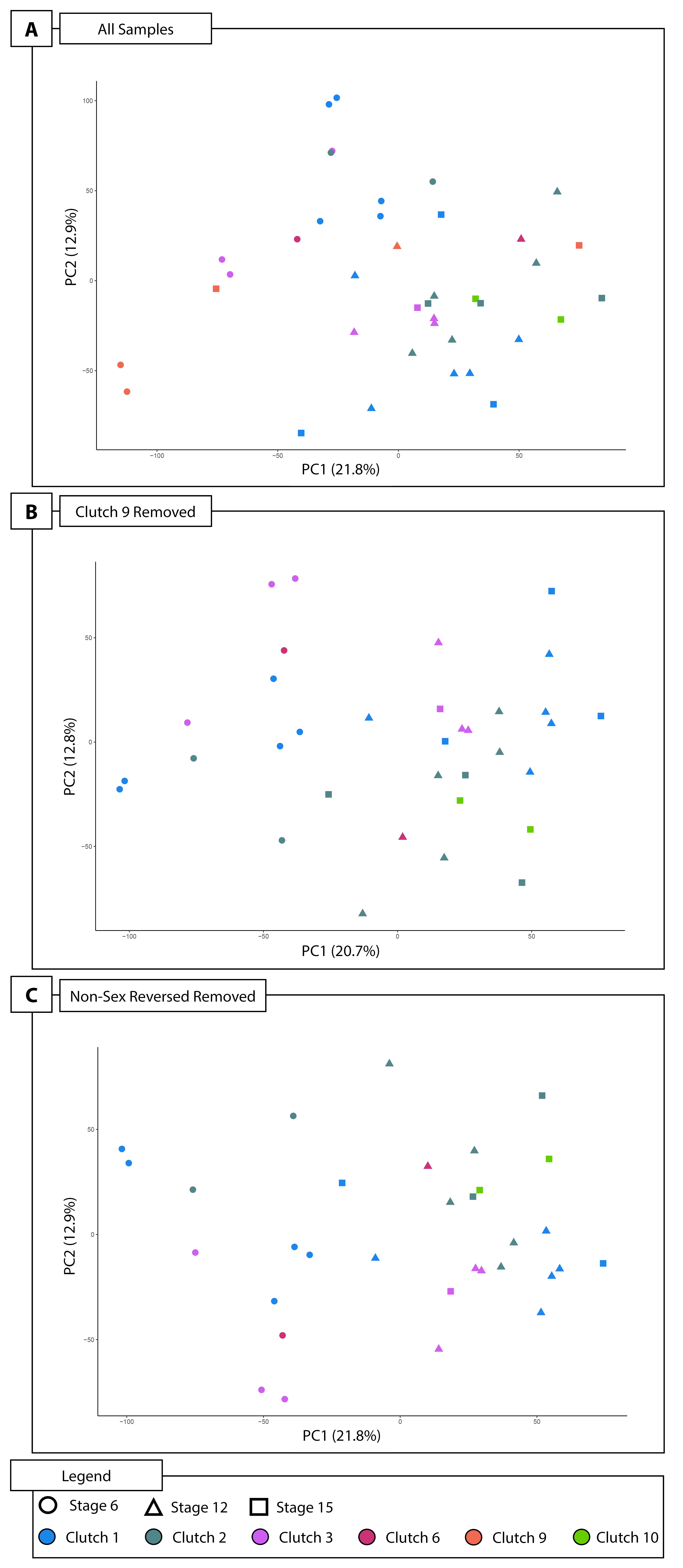

Supplement: S4 Fig — (A) PCA of all samples (n = 39) (B) PCA of samples with clutch 9 removed (n = 32) (C) PCA of samples with clutch 9 samples removed and two samples that had not undergone sex reversal. This is the final dataset upon which all analysis was performed (n = 30). (TIF) [file pgen.1009465.s015.tif]
